# Casual associations between brain structure and sarcopenia: A large‐scale genetic correlation and mendelian randomization study

**DOI:** 10.1111/acel.14252

**Published:** 2024-06-17

**Authors:** Guang Yang, Wenqing Xie, Bin Li, Guihu Zhao, Jinchen Li, Wenfeng Xiao, Yusheng Li

**Affiliations:** ^1^ Department of Orthopedics Xiangya Hospital, Central South University Changsha Hunan China; ^2^ Bioinformatics Center Xiangya Hospital, Central South University Changsha Hunan China; ^3^ National Clinical Research Center for Geriatric Disorders, Department of Geriatrics, Xiangya Hospital Central South University Changsha Hunan China; ^4^ Department of Neurology Xiangya Hospital, Central South University Changsha Hunan China; ^5^ Center for Medical Genetics & Hunan Key Laboratory of Medical Genetics, School of Life Sciences Central South University Changsha Hunan China

**Keywords:** BIDPs, brain, LDSC, mendelian randomization, sarcopenia

## Abstract

Sarcopenia presenting a critical challenge in population‐aging healthcare. The elucidation of the interplay between brain structure and sarcopenia necessitates further research. The aim of this study is to explore the casual association between brain structure and sarcopenia. Linkage disequilibrium score regression (LDSC) was conducted to estimate the genetic correlations; MR was then performed to explore the causal relationship between Brain imaging‐derived phenotypes (BIDPs) and three sarcopenia‐related traits: handgrip strength, walking pace, and appendicular lean mass (ALM). The main analyses were conducted using the inverse‐variance weighted method. Moreover, weighted median and MR–Egger were conducted as sensitivity analyses. Genetic association between 6.41% of BIDPs and ALM was observed, and 4.68% of BIDPs exhibited causal MR association with handgrip strength, 2.11% of BIDPs were causally associated with walking pace, and 2.04% of BIDPs showed causal association with ALM. Volume of ventromedial hypothalamus was associated with increased odds of handgrip strength (OR: 1.18, 95% CI: 1.02 to 1.37) and ALM (OR: 1.05, 95% CI: 1.01 to 1.09). Mean thickness of G‐pariet‐inf‐Angular was associated with decreased odds of handgrip strength (OR: 0.83, 95% CI: 0.70 to 0.97) and walking pace (OR: 0.97, 95% CI: 0.93 to 0.99). As part of the brain structure forward causally influences sarcopenia, which may provide new perspectives for the prevention of sarcopenia and offer valuable insights for further research on the brain‐muscle axis.

## Graphical abstract

In this study, 1325 BIDPs and 5 sarcopenia‐related traits were included. A genetic association between 6.41% of BIDPs and ALM was observed. VMH was associated with increased odds of handgrip strength (OR: 1.18, 95% CI: 1.02 to 1.37) and ALM (OR: 1.05, 95% CI: 1.01 to 1.09); mean thickness of G‐pariet‐inf‐Angular was associated with decreased odds of handgrip (OR: 0.83, 95% CI: 0.70 to 0.97) and walking pace (OR: 0.97, 95% CI: 0.93 to 0.99). This study may offer valuable insights for further research on the brain‐muscle axis.

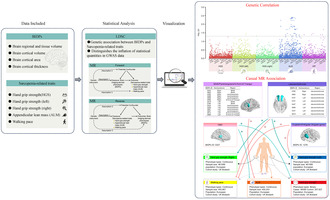

**Abbreviations**
ALM: appendicular lean mass
BIDPs: brain imaging‐derived phenotypes
GWAS: genome‐wide association study
h^2^
: heritability
HGS: hand grip strength
IVW: inverse‐variance weighted
LDSC: linkage disequilibrium score regression
MAF: minor allele frequency
MR: mendelian randomization
MRI: magnetic resonance imaging
OR: odd ratio
SNPs: single nucleotide polymorphisms
VMH: volume of ventromedial hypothalamus
WP: walking pace

## 1 INTRODUCTION

Sarcopenia is a progressive, systemic skeletal muscle disease characterized by accelerated loss of muscle mass, muscle strength, and/or physical performance, which is associated with increased risks of falls, functional decline, frailty, and mortality (Cruz‐Jentoft & Sayer, 2019). There is significant variation in the prevalence of sarcopenia among different regions and ethnicities. Data released by the Asian Working Group for Sarcopenia (AWGS) indicate that the prevalence of sarcopenia among older adults in Asia is approximately 5.5% to 25.7% (Chen et al., 2020). Data from the European Working Group on Sarcopenia in Older People (EWGSOP) reveals that the prevalence of sarcopenia in European communities ranges from 1% to 29%, while in nursing homes, it is between 14% and 33% (Cruz‐Jentoft et al., 2019; Sakuma et al., 2017). Research has shown that the prevalence of sarcopenia in the population aged 65 years and older can reach up to 15%, and in the elderly population aged 80 years and older, the prevalence can be as high as 50% (Cohen et al., 2015). With the escalating global population aging, it is projected that the number of individuals affected by sarcopenia worldwide will rise to half a billion by 2050, leading to increased hospitalization risks and a growing burden of healthcare costs among the elderly (Antunes et al., 2017).

Previous clinical studies have reported an association between brain structure and sarcopenia. Yusuke Osawa's longitudinal study revealed that brain volume changes are associated with changes in muscle strength in older adults (Osawa et al., 2021). Another cross‐sectional study reported that brain CT can predict low lean mass in the elderly with cognitive impairment (Chen et al., 2022). These studies suggest that brain structure may have potential influence on sarcopenia. However, contrasting results have also been reported. In a study of 1284 subjects, sarcopenia was linked to a reduction in gray matter volume, with a pronounced effect in the parietal lobes (Yu et al., 2021). A large‐scale study based on the UK Biobank data found significant associations between sarcopenic traits and both brain structure and cognitive performance; structural changes mediated the relationship between sarcopenia and cognitive traits (Gurholt et al., 2024). These observational studies are mostly based on cross‐sectional study or case–control study, with limited sample sizes due to research cost or medical ethics constraints, making it difficult to adequately adjust for confounding factors between groups (such as lifestyle, occupation, or educational level). The conclusions drawn from the brain findings related to sarcopenia are prone to bias. Therefore, high‐quality research designs are still needed to verify the causal relationship.

Mendelian randomization (MR) is a statistical model that utilizes genetic variations as instrumental variables and has been widely applied in assessing causal associations between exposures and outcomes in recent years. It is a powerful approach for exploring risk factors with causal effects on disease occurrence (Hemani et al., 2018; Lawlor et al., 2008). MR is an intermediate type between traditional epidemics and randomized controlled trials, and the level of evidence is somewhere in between. Compared to case–control and cohort studies, MR is characterized by a higher level of evidence (Arsenault, 2022). MR utilizes genetic variations as proxies for exposure factors and conducts randomization within a large population sample to obtain summary statistics of the associations between genetic variations and the outcome phenotype of interest. It estimates the strength of the exposure‐outcome association using a genetic epidemiological model, which reduces the influence of confounding factors and avoids reverse causation inference. Linkage disequilibrium score regression (LDSC) is another classical tool for genetic correlation analysis (Bulik‐Sullivan et al., 2015). LDSC distinguishes the inflation of statistics caused by polygenic genetic architecture from population stratification or other confounding correlation effects. It can be further applied to estimate the genetic correlation between single nucleotide polymorphisms (SNPs) and traits.

In this study, we performed genetic correlation analysis using a GWAS dataset of brain imaging‐derived phenotypes (BIDPs) and sarcopenia‐related traits (handgrip, appendicular lean mass (ALM), and walking pace). We estimated the causal effect sizes of BIDP changes on the risk of sarcopenia using the forward MR model, and the robustness of the results was supplemented by sensitivity analysis and LDSC. To comprehensively assess the causal relationship between changes in brain structure and sarcopenia, reverse MR model was used to consider the possible reciprocal effects that sarcopenia‐related traits have on identified BIDPs. Our findings provide new insights into the brain‐muscle axis and offer potential strategies for predicting and intervening in sarcopenia at the level of brain imaging.

## 2 METHODS AND MATERIALS

### 2.1 Data source

#### 2.1.1 Brain imaging‐derived phenotypes (BIDPs) data

A recent genome‐wide association study (GWAS) was conducted on a population of 33,224 individuals of European ancestry in the UK Biobank to investigate brain imaging traits. Summary statistics for the brain imaging‐derived phenotypes (BIDPs) were obtained from the Oxford Brain Imaging Genetics (BIG40) web server (https://open.win.ox.ac.uk/ukbiobank/big40). A total of 1325 brain imaging structure‐derived phenotypes (BIDPs) were collected from multimodal brain imaging for further analysis. These BIDPs comprised 647 phenotypes associated with brain regional and tissue volume measured using magnetic resonance imaging (MRI), 372 phenotypes related to cortical area assessed by MRI, and 306 phenotypes related to cortical thickness also measured using MRI; all these BIDPs were corrected for age and sex as covariates. Each BIDPs data vector was quantile normalized, resulting in it being Gaussian distributed, with mean = 0 and s.d. = 1 (Smith et al., 2021).

#### 2.1.2 Sarcopenia‐related traits data

Currently, there is no published GWAS study specifically related to sarcopenia. Therefore, we selected summary GWAS data for phenotypes associated with sarcopenia (Chen et al., 2020), including handgrip strength, walking pace, and appendicular lean mass (ALM), to serve as the outcome variables for our Mendelian randomization analysis. (1) Handgrip strength: In this study, we selected a GWAS of grip strength in individuals of European ancestry. The GWAS summary data for grip strength were obtained from the IEU Open GWAS Project (GWAS ID: ukb‐b‐10,215, ukb‐b‐7478) (Elsworth et al., 2020). In the IEU Open GWAS Project, grip strength was treated as a continuous variable. These GWAS studies were conducted based on UK Biobank, and the result were set free in IEU Open GWAS Project. Hand dynamometers in use at UK Biobank assessment centers were calibrated externally and measured in kilograms. However, we recognize that a single unit change in grip strength may not accurately capture the extent of muscle weakness that meets the diagnostic threshold for sarcopenia. To address this limitation, we also incorporated data from another GWAS study in which grip strength was categorized based on the diagnostic thresholds for sarcopenia outlined in the guidelines set by the European Working Group on Sarcopenia in Older People (EWGSOP) (Cruz‐Jentoft et al., 2019). This allowed us to consider grip strength as a categorical variable, providing a more comprehensive assessment of muscle strength in relation to sarcopenia. (2) Walking pace: GWAS summary data for walking pace was obtained from a GWAS study based on the UK Biobank cohort. Self‐reported walking pace was ascertained using the ACE touchscreen question. The GWAS were corrected by age, sex, BMI, genotyping array, and the first 20 principal components of ancestry (Timmins et al., 2020). (3) ALM: The data on appendicular lean mass (ALM) was sourced from a GWAS study conducted by Pei et al. in 2020, based on the UK Biobank cohort. This study had the largest sample size to date, consisting of 450,243 individuals. ALM was defined as the sum of non‐fat mass in both arms and legs. The results were adjusted for limb fat mass, age, and gender during the analysis.

### 2.2 Single‐nucleotide polymorphism (SNP) inclusion and exclusion criteria

To ensure the accuracy and authenticity of the MR results, the following quality control processes were used to select the appropriate IVs. First, SNPs that were significantly related (*p* < 5 × 10^−8^) to the BIDPs were selected as the IVs. Second, the minor allele frequency (MAF) threshold of the SNPs was 0.01. Third, the SNPs with *F* values less than 10 indicated a weak instrument and were excluded. Fourth, since linkage disequilibrium (LD) is a major cause of bias affecting the results of Mendelian randomization studies, it is very important to eliminate the genetic variation of linkage disequilibrium to improve the reliability of research results (Aissani, 2014). A clumping process (*r* ^2^ > 0.001, clumping distance = 10,000 kb) was conducted to assess the LD between the included SNPs. Fifth, SNPs directly associated with sarcopenia‐related traits (*p* < 5 × 10^−8^) were excluded. Palindromic and duplicated SNPs were removed during the harmonization process.

### 2.3 Genetic correlations analysis

Linkage Disequilibrium Score Regression (LDSC) is a tool for analyzing genetic correlations. LDSC distinguishes the inflation of statistical quantities caused by the polygenic genetic basis from population stratification or other confounding effects, enabling the estimation of genetic correlations between SNP‐based heritability and traits. In this study, LDSC was employed as a supplementary approach to quality control GWAS summary data and examine the genetic associations among traits. LDSC allows us to calculate the overall heritability (h^2^), Genomic Inflation Factor (Lambda GC), Intercept, and Mean Chi^2^ for single GWAS summary data, as well as assess the genetic correlation between two GWAS summary data. A significant deviation of Lambda GC from one indicates the presence of population stratification in the GWAS summary data.

### 2.4 MR study design and statistical analysis

**FIGURE 1:**
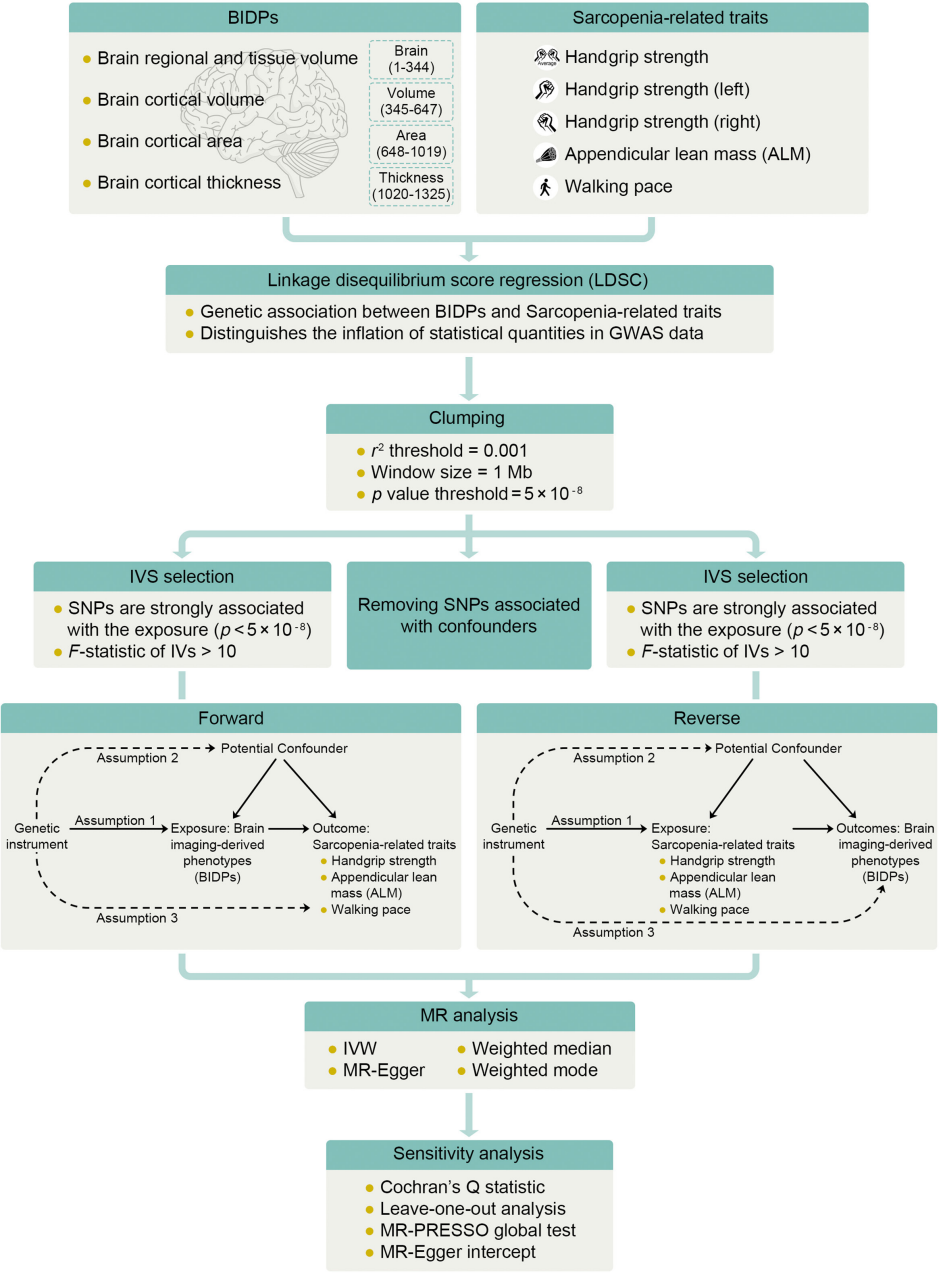
Workflow of two‐sample bidirectional mendelian randomization. Assumption 1: the instrumental variables must be strongly associated with the exposures; Assumption 2: the instrumental variables must be independent of the potential confounders of the association between the exposure and outcome; Assumption 3: the instrumental variables should not be associated with the outcomes directly.

This is a 2‐sample bidirectional Mendelian randomization using genetic variants to mimic the effect of BIDPs on Sarcopenia‐related traits. MR analysis was performed based on the establishment of three core assumptions (Figure 1). To conduct MR estimates, the random‐effects model inverse‐variance weighted (IVW) method was applied as the primary statistical analysis approach (Lee et al., 2016). For the second assumption of MR, we searched the Phenoscanner database to identify whether there were SNPs associated with confounding factors (BMI, Diabetes, Obesity). The weighted median method and MR Egger regression method were also performed as sensitivity analyses.

In this MR study, Cochran's *Q* statistic was used to check the heterogeneity among SNPs in IVW and MR Egger, and the MR‐PRESSO test was used to evaluate overall horizontal pleiotropy. We applied the leave‐one‐out method to examine whether each SNP causes drive or bias in the summary estimates by eliminating each SNP and calculating the meta‐effect of the remaining SNPs. Only findings that have passed the heterogeneity and horizontal pleiotropy tests will be included in this study. All analyses were performed in R software (version 4.3.2). The R packages “TwoSampleMR” (version 0.5.8) and were used to conduct Mendelian randomization. Benjamini–Hochberg procedure implemented in R 3.5.3 was used to obtain adjusted p values; *p* < 0.05 was considered as statistically significant in genetic correlation and MR analyses.

## 3 RESULTS

### 3.1 LDSC results

**FIGURE 2:**
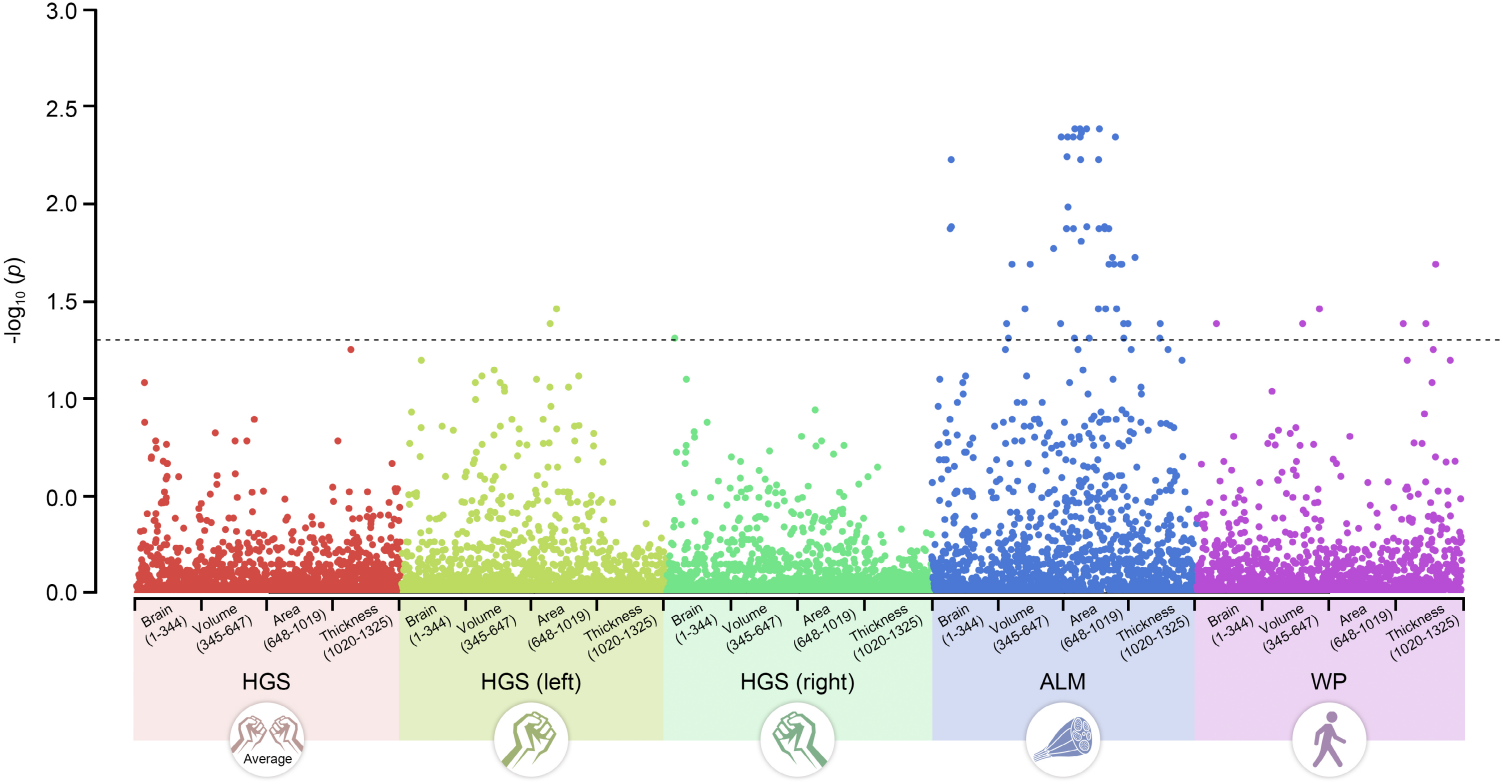
The genetic correlation between BIDPs and Sarcopenia‐related traits. ALM, appendicular lean mass; HGS, handgrip strength; HGS (left), handgrip strength (left); HGS (right), handgrip strength (right); WP, walking pace.

We conducted single‐trait LDSC analysis using GWAS summary data to detect inflation of statistical quantities of exposures and outcomes. The LDSC results revealed that, among all BIDPs, with the exception of 0180 (Volume of non‐WM hypointensities in the whole brain generated by subcortical volumetric segmentation), which exhibited a total heritability of less than 0 (−0.0052), the heritability of the remaining phenotypes consistently exceeded 0 (Table S1). The maximum heritability value observed was 0.284, with an average of 0.1143. Among the sarcopenia‐related phenotypes, ALM displayed the lowest heritability of 0.1821, while walking pace exhibited the highest heritability of 0.7019. The lambda GC values for all five sarcopenia‐related phenotypes were approximately 1 (Table S2). We further explored the genetic correlations between GWAS datasets related to the five sarcopenia‐related phenotypes and 1325 BIDPs using LDSC. The results indicated that, in general, there were no significant genetic correlations between BIDPs and grip strength‐related phenotypes (low handgrip strength, handgrip strength (left), and handgrip strength (right)). However, significant genetic correlation was observed between 0743 (area of superior frontal in the left hemisphere generated by parcellation of the pial surface using Desikan–Killiany parcellation) and 0775 (area of rostral middle frontal in the right hemisphere generated by parcellation of the pial surface using Desikan–Killiany parcellation) with handgrip strength (left). Notably, ALM exhibited significant genetic correlations with multiple BIDPs (Figure 2 and Table S3). In contrast, only six BIDPs (0099, 0409, 0614, 0905, 1146, and 1195) showed genetic correlations with walking pace.

### 3.2 Causal MR associations between BIDPs and handgrip strength

**FIGURE 3:**
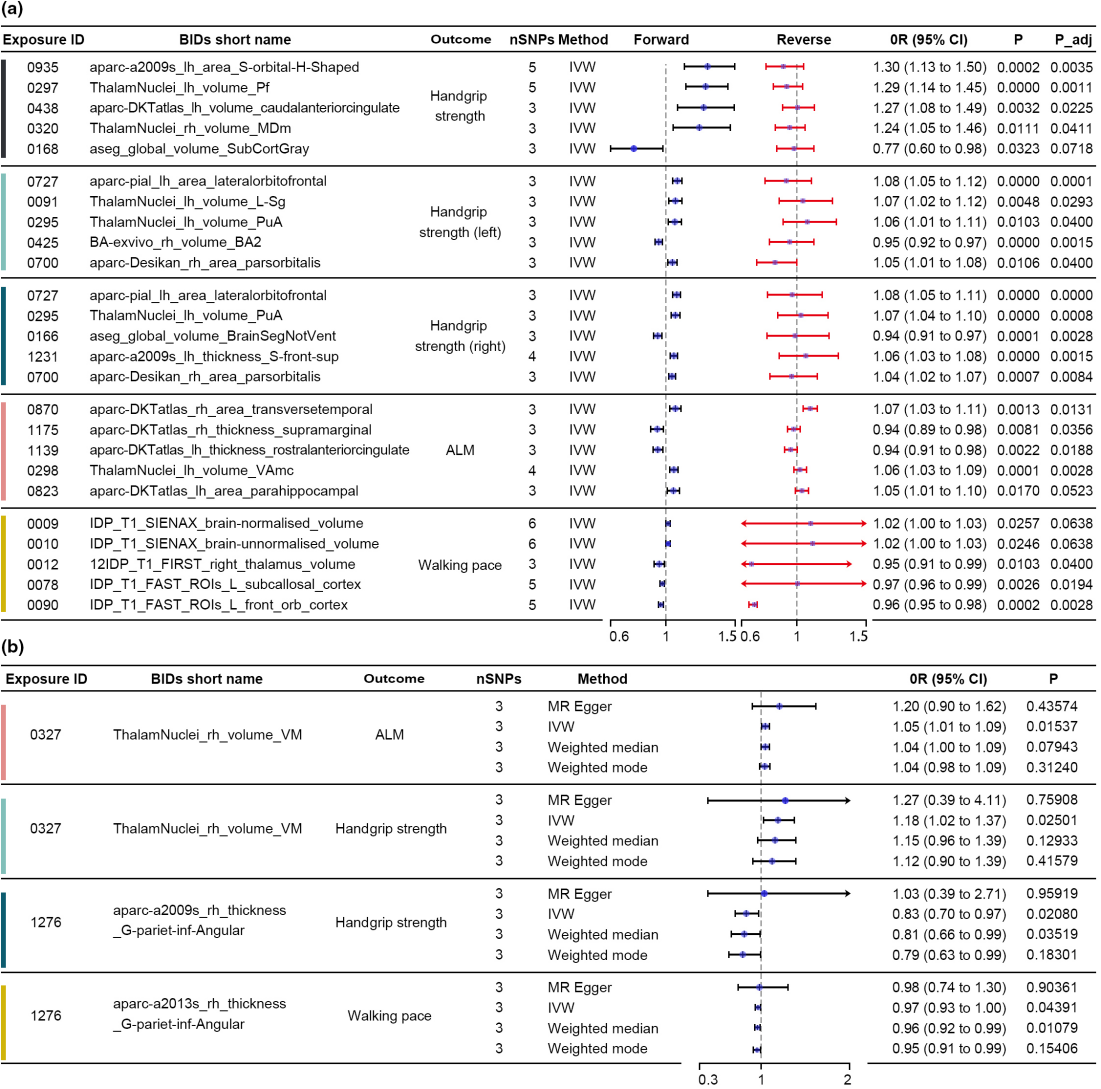
Forest plot of MR results. (a) Bidirectional mendelian randomization results of the top 5 correlations of the BIDPs to sarcopenia‐related traits. (b) Forest plot of forward MR results of 0327 and 1276. ALM: appendicular lean mass.

A total of 62 BIDPs exhibited forward MR casual relationships with grip strength phenotypes, with 27 BIDPs associated with handgrip strength, 17 BIDPs associated with handgrip strength (left), and 18 BIDPs associated with handgrip strength (right) (Table S4). The MR results for the top five BIDPs with the largest absolute effect sizes (OR‐1) on the three grip strength phenotypes are shown in Figure 3a. For handgrip strength, the BIDPs with the largest effects were 0935 (OR: 1.3, 95% CI: 1.13 to 1.50), followed by 0297 (OR: 1.29, 95% CI: 1.14 to 1.45), 0438 (OR: 1.27, 95% CI: 1.08 to 1.49), 0320 (OR: 1.24, 95% CI: 1.05 to 1.46), and 0168 (OR: 0.77, 95% CI: 0.60 to 0.98). For handgrip strength (left), the top five BIDPs ranked by absolute values of OR‐1 were 0727 (OR: 1.08, 95% CI: 1.05 to 1.12), 0091 (OR: 1.07, 95% CI: 1.02 to 1.12), 0295 (OR: 1.06, 95% CI: 1.01 to 1.11), 0425 (OR: 0.95, 95% CI: 0.92 to 0.97), and 0700 (OR: 1.05, 95% CI: 1.01 to 1.08). For handgrip strength (right), the top five BIDPs ranked by the absolute values of OR‐1 were 0727 (OR: 1.08, 95% CI: 1.05 to 1.11), 0295 (OR: 1.07, 95% CI: 1.04 to 1.10), 0166 (OR: 0.94, 95% CI: 0.91 to 0.97), 01231 (OR: 1.06, 95% CI: 1.03 to 1.08), and 0700 (OR: 1.04, 95% CI: 1.02 to 1.07). BIDPs 0295, 0700, and 0727 exhibited effects in the same direction odds on grip strength in both hands. The reverse MR analysis did not identify statistically significant results.

### 3.3 Causal MR associations between BIDPs and walking pace

**FIGURE 4:**
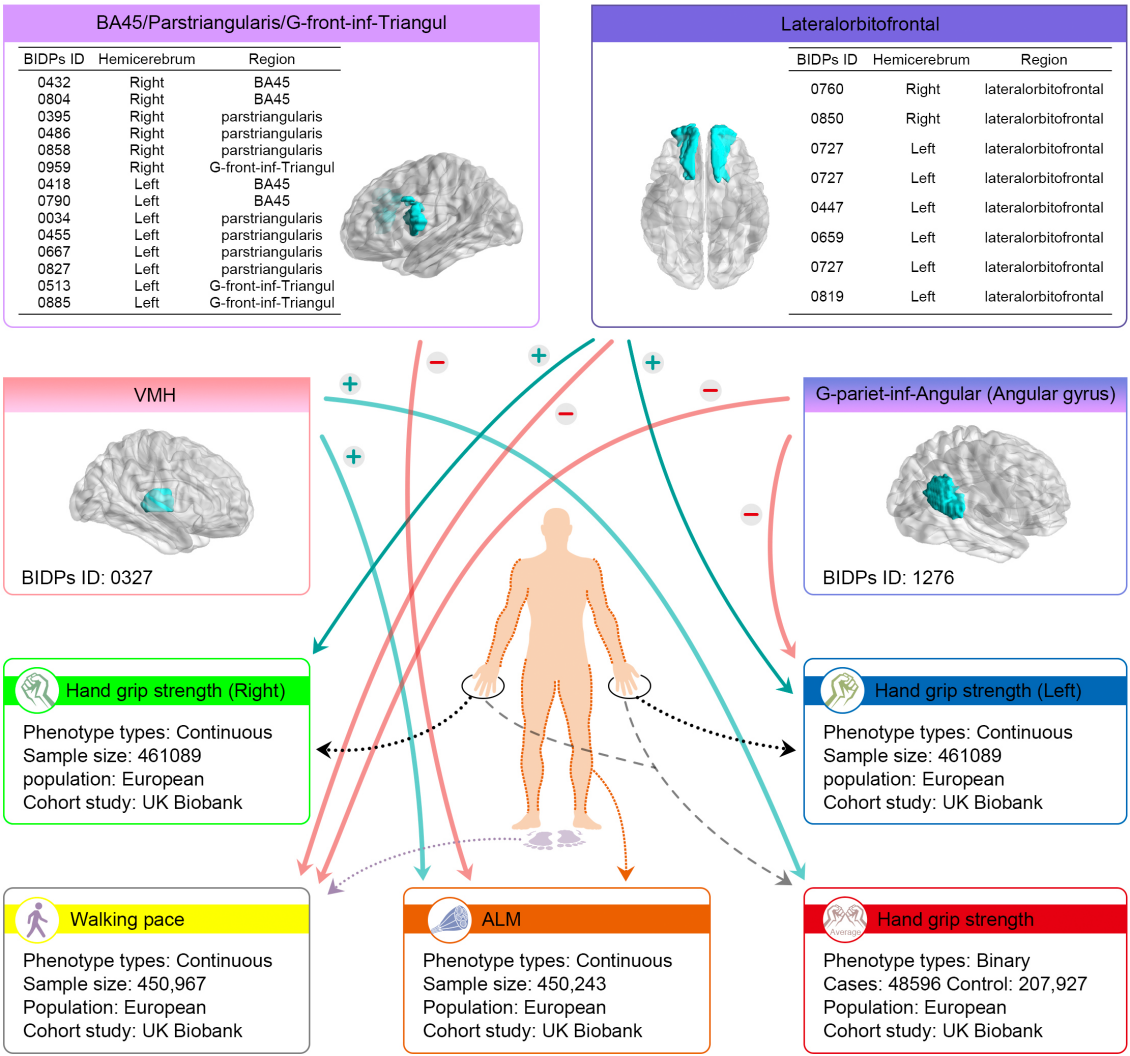
Interaction pattern between BIDPs and sarcopenia‐related traits. ALM, appendicular lean mass; VMH, Volume of ventromedial hypothalamus.

A total of 28 BIDPs exhibited causal MR association with walking pace. Among them, the top five BIDPs, based on the absolute values of OR‐1, were as follows: 0009 (OR: 1.02, 95% CI: 1.00 to 1.03), 0010 (OR: 1.02, 95% CI: 1.00 to 1.03), 0012 (OR: 0.95, 95% CI: 0.91 to 0.99), 0078 (OR: 0.97, 95% CI: 0.96 to 0.99), and 0090 (OR: 0.96, 95% CI: 0.95 to 0.98) (Figure 3a). Notably, among these significantly associated BIDPs, multiple findings indicate that lateralorbitofrontal (0447, 0659, 0727, 0760, 0819, 0850) (Figure 4) and transversetemporal (0680, 0839, 0870) have potential interaction with walking pace. Specifically, the volume of the lateralorbitofrontal region was associated with decreased odds of walking pace, while the volume of the transversetemporal region was associated with increased odds of walking pace (Table S4).

### 3.4 Causal MR associations between BIDPs and ALM

A total of 27 BIDPs exhibit significant MR causal relationships with ALM, and 0870 was excluded due to its genetic correlation with ALM. Among these BIDPs significantly associated with ALM, the top five based on the absolute values of OR‐1 are 0870 (OR: 1.07, 95% CI: 1.03 to 1.11), 1175 (OR: 0.94, 95% CI: 0.89 to 0.98), 1139 (OR: 0.94, 95% CI: 0.91 to 0.98), 0298 (OR: 1.06, 95% CI: 1.03 to 1.09), and 0823 (OR: 1.05, 95% CI: 1.01 to 1.10) (Figure 3a). MR results consistently suggest that the parstriangularis regions (0034, 0395, 0455, 0486, 0667, 0827, 0858) appear to have the closest relationship with ALM, followed by the BA45 region (0418, 0432, 0790, 0804) (Table S4 and Figure 4).

### 3.5 BIDPs with multi‐MR effects in sarcopenia‐related traits

Table S5 provides a list of BIDPs that shows significant MR causal relationships with multiple sarcopenia phenotypes. Among them, 0700 and 0727 are associated with three sarcopenia‐related phenotypes. These BIDPs were associated with increased odds of handgrip strength (left and right) and decreased odds of walking pace. Forward MR analysis revealed significant causal relationships between 0700 and multiple variables, including handgrip strength (left) (OR: 1.05, 95% CI: 1.04 to 1.06), handgrip strength (right) (OR: 1.04, 95% CI: 1.03 to 1.05), and ALM (OR: 0.96, 95% CI: 0.95 to 0.97). For 0727, the OR are 1.08 (95% CI: 1.07 to 1.09) to handgrip strength (left), 1.08 (95% CI: 1.07 to 1.09) to handgrip strength (right), and 0.96 (95% CI: 0.95 to 0.98) to ALM, and 0327 was associated with increased odds of handgrip strength (OR: 1.18, 95% CI: 1.02 to 1.37) and ALM (OR: 1.05, 95% CI: 1.01 to 1.09). 1276 (Mean thickness of G‐pariet‐inf‐Angular) was associated with decreased odds of handgrip strength (OR: 0.83, 95% CI: 0.70 to 0.97) and walking pace (OR: 0.97, 95% CI: 0.93 to 0.99). (Figure 3b, Figure 4).

## 4 DISCUSSION

The rapid development of brain imaging techniques, represented by MRI, enables in vivo quantitative assessment of human brain structure, function, connectivity, and network characteristics, thereby obtaining a series of brain imaging phenotypes. Based on high‐resolution structural MRI, it is possible to extract measurements such as whole brain volume, gray matter volume, white matter volume, and subcortical nucleus volume, as well as the volume, thickness, and surface area of individual cortical brain regions (Gong et al., 2021). The cerebral cortex, which is the outer gray matter layer of the brain in the human body, serves as the foundation for complex cognitive abilities and involves various aspects, including higher‐order cognitive functions (Shafee et al., 2015). During embryonic development, the main neuronal cell type in the cortex, excitatory neurons, is generated from neural progenitor cells in the germinal zone. The expansion of the cortical surface area (SA) is driven by the proliferation of these neural progenitor cells, while the number of neurogenic divisions determines its thickness (TH) (Thompson et al., 2020). The measured values of SA and TH can effectively reflect the characteristics of the cerebral cortex and exhibit a high degree of heritability (Grasby et al., 2020). Of the structural MRI IDPs, volumetric measures are the most heritable and cortical thicknesses the least (Elliott et al., 2018).

Sarcopenia, along with neurodegenerative diseases such as Parkinson's disease and Alzheimer's disease, represents manifestations of geriatric syndrome. Regardless of the study population and the criteria for defining sarcopenia and neurodegenerative diseases, both exhibit a relatively stable correlation (Hart et al., 2023; Yang et al., 2022; Ye et al., 2023). However, a study has also shown no relationship between sarcopenia and cortical brain structure. This is mainly due to the fact that limb muscle mass was not included in the diagnosis of the participants, and not all patients underwent grip strength and timed up and go (TUG) measurements, which may lead to misdiagnosis of sarcopenia (Hassan et al., 2019).

This study found that the area of lateral orbitofrontal in the left hemisphere, volume of PuA in the left hemisphere, and area of parsorbitalis in the right hemisphere have effects in the same direction (positive or negative) on handgrip strength of both hands. The impact of lateral orbitofrontal cortex surface area is the greatest, positively influencing handgrip strength of both hands. Previous studies have indicated the presence of effective connections between the orbitofrontal cortex (medial and lateral) and the posterior cingulate gyrus, including the precuneus. These regions appear to have the ability to influence each other and do not function as separate systems (Rudebeck & Rich, 2018). The orbitofrontal cortex projects to the medial cingulate gyrus and other motor prefrontal cortical regions, providing action‐outcome learning, including limb withdrawal or opposition to aversive and non‐rewarding stimuli. Other visual neurons in the orbitofrontal cortex respond to facial expressions and gestures with emotional and social signaling value. These inputs may be received from the superior temporal sulcus region, where neurons respond to these types of facial expressions and motion stimuli (Rolls et al., 2022). These findings can partially explain the positive impact of lateral orbitofrontal cortex surface area on handgrip strength of both hands. For ALM, this study found a negative correlation between the volume of the parstriangularis, including grey matter in the left inferior frontal gyrus, parstriangularis in the right hemisphere, parstriangularis in the left hemisphere, and Brodmann Area 45, and ALM. Although there is no direct research evidence demonstrating a direct relationship between the volume of the parstriangularis and muscle mass, some studies suggest that changes in brain structure may be associated with changes in muscle mass (Kilgour et al., 2013; Muchlinski et al., 2018). The structure and function of the brain can be influenced by external environmental factors. For example, factors such as malnutrition and lack of exercise may lead to changes in brain structure, indirectly affecting muscle mass (Sui et al., 2020). Therefore, further research is needed to better understand the relationship between these two factors. This study also found a negative correlation between the volume of the lateral orbitofrontal cortex and walking pace and a positive correlation between the volume of the transverse temporal gyrus and walking pace. The relationship between the volume of the lateral orbitofrontal cortex and walking pace, as well as the volume of the transverse temporal gyrus and walking pace, has not been sufficiently studied and requires further exploration through neuroimaging and kinematic research.

Furthermore, this study also found that the volume of the ventromedial hypothalamus (VMH) in the right cerebral hemisphere and the average thickness of the angular gyrus region in the right cerebral hemisphere exhibit the same direction of OR in different phenotypes of sarcopenia. Specifically, the volume of the ventromedial thalamus is positively correlated with muscle strength and muscle mass, while the average thickness of the angular gyrus region is negatively correlated with muscle strength and physical performance. According to the 2019 AWGS consensus, a decrease in muscle strength and muscle mass is defined as “sarcopenia,” while a decrease in muscle strength and/or physical performance is defined as “possible sarcopenia” (Chen et al., 2020). Recent studies have found that the VMH may be a critical neural structure involved in coordinating arousal levels and motor function. On one hand, VMH neurons can send excitatory projections to the anterior lateral motor cortex, influencing the sustained firing activity of motor cortex neurons and thus participating in motor preparation and initiation (Guo et al., 2017; Takahashi et al., 2021). On the other hand, VMH neurons can also project to widespread areas of the cerebral cortex, regulating the excitatory activity of superficial neurons in the cerebral cortex (Guo et al., 2018; Kuramoto et al., 2015), thus enhancing the level of arousal in the organism (Honjoh et al., 2018). In addition, VMH neurons receive signal inputs from the medial prefrontal cortex, motor cortex, striatum, and cerebellum (Guo et al., 2017; Kebschull et al., 2020; Tanaka et al., 2018). The latest research also suggests that specific projections to the VMH from deep cerebellar nuclei (DCN) neurons can play a role in amplifying sensorimotor learning responses by regulating the activity of the parietal cortex (Zhang et al., 2022). Therefore, the VMH is in a favorable anatomical position that allows it to integrate information related to body movement and proprioception. It can then coordinate the level of arousal and motor function through axonal projections. This study also provides evidence from the perspective of common genomic variations and genetic correlations that the volume of the VMH is positively correlated with muscle strength and muscle mass, serving as a protective factor against the occurrence of sarcopenia. The angular gyrus is involved in numerous cognitive tasks, including semantic processing, spatial cognition, memory retrieval, attention, as well as word reading and comprehension (Rockland, 2023). In addition, the angular gyrus is part of the default network and is considered important in cognitive impairment associated with neurodegenerative diseases. Although there is currently no direct research on the relationship between angular gyrus cortical thickness and sarcopenia‐related features, multiple studies have found that thinning of the angular gyrus cortical thickness plays a significant role in the onset of neurodegenerative diseases. A study utilizing machine learning investigated whether MRI information on cortical thickness can aid in predicting the individual‐level progression from Parkinson's disease (PD) and mild cognitive impairment (MCI) to Parkinson's disease dementia (PDD). They found that, within the cortical thickness features, thinning of the left angular gyrus cortex was the most common observation, with high importance and weight. Therefore, the functional impairments in the posterior cortical regions and frontal lobe, which serve as neurocognitive predictors of PDD conversion, may be partially attributed to angular gyrus atrophy (Shin et al., 2021). Another study also indicated that compared to the cognitively normal group, the Alzheimer's disease (AD) dementia group exhibited significant cortical thickness reduction in the bilateral parietal lobe regions, including the angular gyrus. Additionally, compared to the MCI group, the AD dementia group showed significant cortical thickness reduction in several small clusters within the frontal and parietal lobe regions. However, there were no regions with significant cortical thickness reduction in the MCI group compared to the control group (Vogt et al., 2020). Our study also confirmed through MR that thinning of the angular gyrus cortex is a potential risk factor for sarcopenia. However, further validation is still needed through clinical cohorts or case–control studies in the future.

In this study, we encountered a perplexing phenomenon of decreased heritability. and negative estimates. SNP heritability of 7.02% for walking pace was lower than it was reported before (8.2%) (Timmins et al., 2020), and SNP heritability of BIDP 0180 (Volume of non‐WM‐hypointensities in the whole brain generated by subcortical volumetric segmentation) exhibited a total heritability of less than 0 (−0.0052). The possible explanation for this phenomenon is that LD score regression, which utilizes the slope from *χ* ^2^ (from GWAS) regressed on SNPs' LD scores to estimate the heritability due to complex variations (CVs) in LD with common SNPs, often underestimates heritability when the trait is highly polygenic (Tenesa & Haley, 2013).

This study has some limitations. Firstly, the datasets used for MR and LDSC analyses of sarcopenia‐related exposure factors and outcomes are derived from GWAS analyses of European populations. While this reduces bias due to population stratification, it also limits the generalizability of the findings. Genetic structures and disease‐related risk loci may exhibit heterogeneity in populations of Asian, African, and other non‐European ethnicities due to genetic differences. Secondly, MR analysis utilizes genetic instrumental variables to represent exposure factors. Given the limitations of the sample size and additive regression models in the current study, the proportion of variance in the exposure factors that can be explained is still relatively small. Therefore, it is difficult to detect weak causal effects between complex traits. Thirdly, GWAS data for exposures and outcomes were all from the UK Biobank; this could potentially lead to bias due to sample overlaLastly, the effect estimates obtained from genetic correlation analyses can only represent estimated values based on the current dataset and model. They cannot be considered equivalent to or replace the effect estimates obtained from clinical observational studies. Integrating genetic correlation analyses with classical epidemiological analyses, real‐world studies, systematic reviews, or meta‐analyses of the literature is necessary to obtain the best evidence for clinical practice.

## 5 CONCLUSION

The findings demonstrate a significant genetic correlation between ALM and BIDPs, revealing the intricate interplay between BIDPs and sarcopenia. In addition, our analysis detects the potential prognostic significance of two specific brain region, volume of ventromedial hypothalamus and mean thickness of G‐pariet‐inf‐Angular, in predicting the susceptibility to sarcopenia. As part of the brain structure forward causally influences sarcopenia, which may provide new perspectives for the prevention of sarcopenia and offer valuable insights for further research on the brain‐muscle axis.

## AUTHOR CONTRIBUTIONS

Conceptualization, GY; methodology, BL and YSL; verification of the underlying data, GY, WQX; writing—original draft, GY, WQX; writing—review and editing, BL, YSL, GHZ, JCL and WFX. All the authors participated in planning, execution, and analysis and have read and approved the final submitted version.

## FUNDING INFORMATION

This work was supported by the National Key R&D Program of China (2021YFC2502100, 2023YFC3603404, 2019YFA0111900), National Natural Science Foundation of China (82072506, 82272611, 92268115), Hunan Provincial Science Fund for Distinguished Young Scholars (2024JJ2089), Hunan Young Talents of Science and Technology (2021RC3025), Provincial Clinical Medical Technology Innovation Project of Hunan (2023SK2024, 2020SK53709), Provincial Natural Science Foundation of Hunan (2020JJ3060), National Natural Science Foundation of Hunan Province (2023JJ30949), National Clinical Research Center for Geriatric Disorders, Xiangya Hospital (2021KFJJ02, 2021LNJJ05), the Hunan Provincial Innovation Foundation for Postgraduate (CX20230308, CX20230312), the Independent Exploration and Innovation Project for Postgraduate Students of Central South University (2024ZZTS0163).

## CONFLICT OF INTEREST STATEMENT

The authors declare that they have no competing interests.

## Supporting information


Tables S1–S6.

## Data Availability

The data that support the findings of this study are openly available in the link blow: BIDPS: https://open.win.ox.ac.uk/ukbiobank/big40. Handgrip strength: https://gwas.mrcieu.ac.uk/, https://www.ebi.ac.uk/gwas/home. Walking pace: https://pubmed.ncbi.nlm.nih.gov/33128006/. Appendicular lean mass (ALM): https://pubmed.ncbi.nlm.nih.gov/33097823/.
